# An Overview on Nettle Studies, Compounds, Processing and the Relation with Circular Bioeconomy

**DOI:** 10.3390/plants13243529

**Published:** 2024-12-17

**Authors:** Ioana-Maria Toplicean, Rebeca-Didina Ianuș, Adina-Daniela Datcu

**Affiliations:** Biology Department, Faculty of Chemistry, Biology, Geography, West University of Timisoara, Pestalozzi J.H. 16, 300115 Timisoara, Romania; ioana.toplicean03@e-uvt.ro (I.-M.T.); rebeca.ianus03@e-uvt.ro (R.-D.I.)

**Keywords:** natural fibers, sustainability, uses in industry, biological active compounds, CBE

## Abstract

This paper provides an interdisciplinary overview of nettle bioactive compounds and processing, and ir also explores its role in the circular bioeconomy. *Urtica dioica* L. is sometimes referred to as a multipurpose herbaceous species that has been used historically in food, textiles, and medicine owing its rich profile of biological compounds. This study synthesizes the recent literature to examine nettle’s applications across various industries, from nutritional supplements to eco-friendly fiber materials. In addition, it highlights nettle’s potential in sustainable production chains, aligning with the EU’s bioeconomy directives. The methods involve a comprehensive literature review and data analysis, with a focus on bioactive compounds and eco-sustainable applications. The results of this review underscore the plant’s unique adaptability to low-input farming and its contributions to reducing resource dependency. The findings position nettle as a valuable resource for sustainable innovation, emphasizing its relevance within circular economic models.

## 1. Introduction

Stinging nettle (*Urtica dioica* L.) is a versatile plant known for its bioactive compounds, with applications in medicine, cosmetics, and textiles. Beyond these functional uses, nettle plays a significant role in promoting sustainable practices aligned with circular bioeconomy principles. Despite its potential, key research gaps remain, such as exploring its scalability for industrial use and its economic viability. Known for its diverse applications, it plays a significant role in sustainable industries and the circular bioeconomy. It creates thickets where it thrives, especially in disturbed areas [1]. In phytotherapy, its derivatives—such as crude dried powder, decoction (herbal tea), dry extract or virgin juice—are clearly significant [2]. The plant species is a member of the phytoalimurgic vegetable group. This comprises the edible wild species that were utilized in the past during times of food scarcity. The phrase comes from the Latin terms alimenta urgentia, which means any type of food that is accessible in an emergency. For therapy of rheumatoid arthritis or osteoarthritis symptoms, as well as for enhanced diuresis, such as in the event of urinary tract infections, raw or dehydrated leaves or blooming aboveground parts of *Urtica dioica* L., their hybrids, or combinations of these are advised [3].

It is commonly known that *U. dioica* causes annoyance when handled. It injects an irritating liquid in a manner similar to that of a hypodermic needle, resulting in welts, red pimples, and skin irritation (the word “nettle” comes from an Anglo-Saxon word meaning “needle”). The mechanical and metabolic functions of the plant are what lead to stinging nettle dermatitis [4]. The plant has long underground shoots, ovate-lanceolate leaves, and axillary inflorescences with tiny greenish flowers. All subspecies are upright perennials, growing up to two meters in height [5,6,7].

Fresh leaves of this plant can be dried and utilized as powder or in various ways, and it has recently gained popularity as a very nutrient-dense food. Numerous bioactive substances, including flavonoids, phenolic acids, and amino acids, are abundant in the leaves [8]. 

At least since the Middle Ages, people have utilized stinging nettle for food and fiber. Because it thrives even in northern latitudes, this plant species, along with flax and hemp, is the most significant botanical-based raw textile material in Europe. During the 19th century, Germany and Austria were the first to cultivate nettles and start commercial nettle fiber farming [9]. In the recent past, there has been a revived attentiveness in reusing organic or biobased fibers due to rising worries regarding the use of perpetual resources in production [10].

Because *U. dioica* is a perennial crop with minimal pesticide and fertilizer requirements [11], it has a high growth potential in numerous places and can be widely cultivated [12]. It might enhance soils that are overloaded with phosphates and nitrates. Because it is herbaceous nitrophilous plant [13], it increases regional fauna and flora diversity [12], and it is harvested from land that is unfit for the production of nourishment, involving contaminated terrains. These are proper ecological and economic causes for growing *Urtica dioica* as a fiber crop as well. 

The species’ high degree of variety and susceptibility to collecting pesticides and heavy metals may be factors in why raw materials from plants that grow in the wild are sometimes of dubious quality [14,15].

Nettle plants can reach two meters in height and are characterized by stinging hairs and protective trichomes, which play a role in herbivore deterrence [16]. They are substantially recognized for their capacity to cause dermatitis if touched. This is due to the hairs’ needle-like release of biological mediators including acetylcholine and histamine [8]. The density of trichomes is lower on the top surface of the leaves, in the internodes, and at the base of the stem [17].

Stinging trichomes, whose density is a heritable trait, protect nettles from vertebrate herbivores [6,7]. In fact, populations in regions with less extensive animal grazing have fewer trichomes than those under intense mammal grazing [18]. For example, in a culture experiment, growing *Urtica dioica* in the shade instead of the light resulted in fewer trichomes, demonstrating the importance of phenotypic plasticity [6]. Certain studies have also suggested that certain differences, such as variations in hair density, seem to be inherited and genetic [7]. 

This work’s main aim is to describe the relation between different types of nettle processing methods and their influence on the circular economy. 

The primary goals are to: Examine the specific literature on pertinent nettle research, paying particular attention to previously published articles from a variety of sources, including PubMed, Web of Science, and Google Scholar; to go into great detail about this plant’s primary biologically active substances; to concentrate on research on nettle fibers and their applications in many industries; and to compile workable ideas for utilizing nettle in the circular economy in compliance with EU regulations. The research goals aim to provide a multidisciplinary perspective on the applications and benefits of nettle.

## 2. Materials and Methods

For the fulfillment of this review’s objectives, a methodical article search was realized using the PRISMA2020, Preferred Reporting Items for Systematic Reviews and Meta-Analyses. The use of such a method assures more transparency through detailed documentation. Moreover, the criteria for the inclusion/exclusion of the studies are mentioned [19]. The steps are the planning of the study, followed by conducting the review investigation and reporting [20].

The first step, already detailed in the Introduction, was the definition of the study aim and objectives, as well as choosing the keywords. In addition, the following exclusion criteria were defined: the text is not accessible as a full text or in an accessible language. Nonetheless, duplicative content was avoided. Concerning the criteria for inclusion, we chose studies matching the analyzed areas, namely uses, biologically active compounds, fibers, bioeconomy and circular economy aspects related to nettle. The second step of this study involved searching for relevant articles. Literature was selected using Google Scholar [21,22], Web of Science [23] and PubMed [24] using keywords such as ‘nettle uses’, ‘bioactive compounds from nettle’ and ‘nettle utilities’. Articles published after 2000 and available in full text were included. Duplicate studies and non-relevant content were excluded. Articles were included based on their relevance to nettle’s bioactive compounds, sustainable cultivation, and industrial applications. The inclusion criteria were as follows: studies published in peer-reviewed journals between 2000 and 2024, available in full text, and written in English. The exclusion criteria included duplicate studies, non-peer-reviewed sources, and articles lacking methodological transparency. Methodological robustness was assessed using PRISMA guidelines. In the second place, an analytical evaluation of the appropriate abstracts of the studies was realized. The text was reviewed and critically analyzed, including 164 review and research articles. Among these (164) studies, the ones that best fit the issue and were obviously relevant were included in this article’s discussion. Several viewpoints, findings and patterns that were thought to be pertinent to this study’s goals were included. 

Additionally, logical figures were produced with the help of a website called VOSviewer [25]. The retrieved data served as the basis for these visual depictions. These charts were made to help readers understand difficult subjects and improve their comprehension. These charts are also effective teaching aids, giving both professionals and non-experts a deeper understanding of our topic. This network approach aids in understanding the myriad connections between the circular economy and various aspects of sustainability. 

Closing the knowledge gap between the real application of complex scientific concepts and the visual depiction of data was the aim. This method encourages greater research and discussion in the field, in addition to helping to spread information. Our ultimate goal was to provide a resource for academics, policymakers, and anybody else with an interest in sustainable energy and the bioeconomy. We believe that these efforts will promote a more comprehensive and informed discussion about the potential and issues around biomass and biofuels in relation to sustainable development generally.

## 3. Results and Discussion

### 3.1. Studies on Nettle

**Figure 1:**
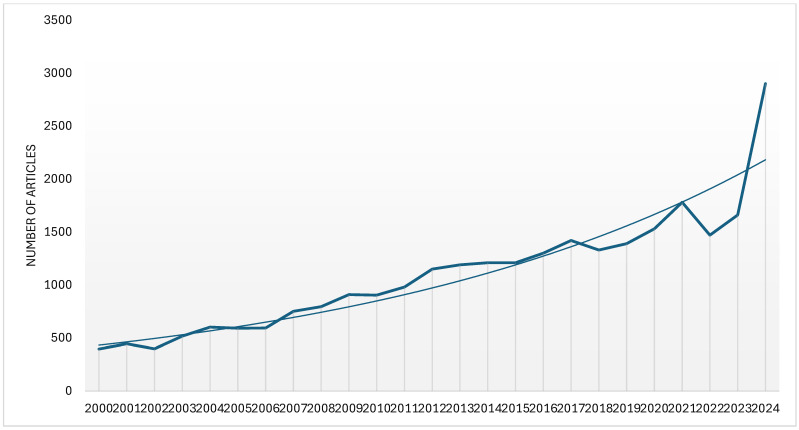
The dynamics of the published studies on Google Scholar concerning *U. dioica* uses between 2000 and 2024 [26].

**Figure 2:**
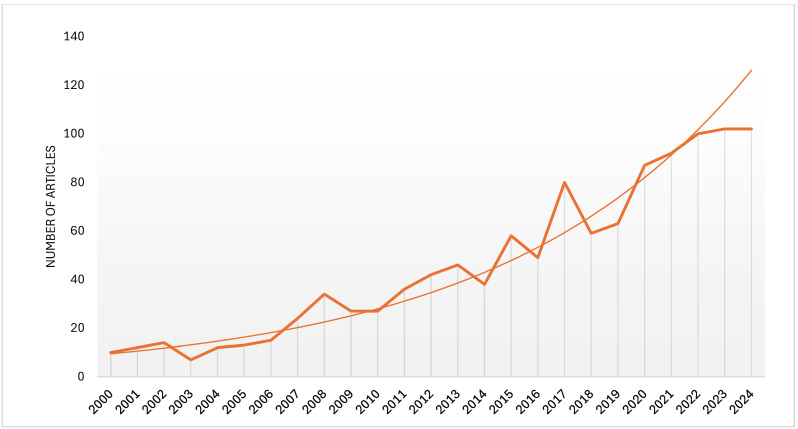
The dynamics of the published studies on Web of Science concerning *U. dioica* uses between 2000 and 2024 [26].

A clear tendency of increasing interest in studies on the utility of the analyzed species was observed. Thus, Figure 1 shows the evolution over time, since 2000, of the number of articles on the usefulness of the nettle, only *U. dioica* species, when we analyzed the data generated by Google Scholar. It can be observed that the researchers have also focused on concepts related to the transition from a linear to a circular economy, looking for solutions in this regard. This fact was also noticed when studies from Web of Science were analyzed (Figure 2).

### 3.2. Main Bioactive Compounds in Nettle

Secondary metabolites, sometimes referred to as bioactive compounds or phytochemicals, are found in the components taken from the roots and aboveground plant parts of medicinal plants. These include a wide range of naturally occurring substances [27], some of these being found in the stinging trichomes, which are particular structures characterized by thick walls with an irritant liquid inside. This hairs act like hypodermic syringes.

It has been demonstrated that nettle, the most prevalent spontaneous perennial herb in the world [28], contains a wide range of chemical components, such as proteins, vitamins, volatile compounds, minerals, terpenes, flavonoids, fatty acids, essential amino acids, polyphenols, tannins, organic acids, lignans, alkaloids, chlorophylls, and polysaccharides [28]. Nettle has been proved to have three times the amount of protein in comparison to other traditional source of proteins, such as rice, barley and wheat [29]. Nettle leaves contain B2, K, C and E vitamins [30,31,32], along with minerals such as manganese, potassium, calcium, phosphorus, magnesium, sodium, copper, zinc, boron, selenium and iron [33,34,35,36,37]. The main categories of triterpenoids and their glycosides are found in their roots and include pentacyclic derivatives of ursane, lupane and oleanane, as well as tetracyclic derivatives of protostane, cycloartane, dammarane and euphane [38,39].

Because its saturated fat is low, its leaves contain an adequate amount of fatty acids, especially palmitic, cis-9,12 linoleic and α-linoleic acids and others such as euric acid, palmitolic acid, stearic acid, behenic acid, dodecendioic acid and tricosanoic acid [30,40,41,42].

Nettle contains 20 amino acids, mostly including the eight essential amino acids except for tryptophan: lysine, isoleucine, histidine, leucine, phenylalanine, methionine, valine and threonine [43,44]. The data indicate that because dried nettle has a higher concentration of amino acids, it has a better amino acid profile than many other green leafy legumes or Brussels sprouts, and it can be a valuable source of amino acids and other nutrients for product development [45,46]. Acids with a phenolic structure, such as gallic acid, caffeic acid, vanillic acid, gentisic acid, syringic acid, protocatechuic acid, caffeic acid, p-coumaric acid, ferulic acid, cinnamic acid, chlorogenic acid and sinapic acid are valuable active compounds found in nettle [47,48,49]. The plant contains flavonoids such apigenin, baicalein, isorhamnetin, amentoflavone, apiin, catechin, epicatechin, epigallocatechin gallate, chrysoeriol, genestein, baicalin, kaempferol, keampferol 3-O-β-d-glucoside and luteolin [30,48,49]. It contains chlorophyll E140, which is used as a food additive [50]. The major carotenoids are β-carotene, lutein isomers, neoxanthin and violaxanthin [51,52]. Formic acid, acetic acid, malic acid, succinic acid and citric acid are the organic acids that were discovered [53]. Polysaccharides such as pectin, hemicellulose and cellulose are also found, representing an important source of dietary fiber [54,55].

Table 1 illustrates the main bioactive compounds that can be found in nettle, along with extraction methods and their uses.

In Table 1, the most abundant chemical groups with their components are highlighted, each of them undergoing unique extraction methods, which can include chromatography-based methods like HPLC and GC-MS or ultrasound-assisted extraction (UAE). These substances have a variety of biological effects that provide a broad range of health advantages, including antimicrobial, anti-inflammatory, antioxidant and anticarcinogenic qualities. 

These substances are useful for skincare, illness prevention and general health enhancement in both the medical and cosmetic domains.

### 3.3. Fibers in Nettle: Extraction Methods

For hundreds of years, stinging nettle has been used to make textiles, being utilized since the 7th century in Switzerland [97]. Also, In Great Britain, the first recorded connection between “nettlecloth” and “Urtica” dates to 1391 [98]. This species, together with flax and hemp, is the most important plant-based textile material in Europe. During the 19th century, Germany and Austria were the first to cultivate nettles and start commercial nettle fiber farming [9]. However, during World War II, nettle processing facilities were destroyed, making other, less expensive fibers more widely accessible [99]. The market for natural fiber-based textiles produced using low-impact methods is growing [100]. Interest in the usage of this plant is attributed to different motives, such as the necessity of finding alternatives to the conventional, unsustainable textile chain, the harm that toxic residues in textiles cause to people’s health, and the rising desire for natural fibers made using low-impact methods that are also less expensive and recyclable [101]. Excellent qualities like high breaking tenacity and flexibility are possessed by nettle fibers [102]. Nettle fibers need less energy to create and are fully biodegradable, just as hemp, kenaf, and coir fibers are. Nettle fibers offer better mechanical and thermal properties than many other natural fibers. Nettle fiber-reinforced thermoplastic composites are being used in automotive interiors and nonstructural applications. Nettle grows without the need for special care, such as fertilizers and pesticides, like hemp fibers. The use of nettle fiber to reinforce plastic aircraft panels and other machine parts has been studied [103]. Higher cellulose contents also make nanocellulose extraction possible, expanding the range of uses for nettle fibers [104]. One type of wild nettle that is grown is fiber nettle. After more than 30 years of breeding, some clones were chosen based on their strong tillering, long, unramified stalks, and high fiber content (up to 16%, as opposed to 4–5% in wild nettle). The most remarkable clones are still kept in German research facilities [105]. The fiber nettle species’ various organs can be utilized as food in biodynamic agriculture, industry, medicine, and cosmetics, or as fodder or raw materials for a variety of purposes [106].

Ropes, fishing nets, silky fabric, cloth, paper, biocomposites, and other materials are made from the fiber tissues of stems [105,107]. The main process of extracting nettle fiber could also be broken down into the following five steps, despite the fact that the extraction techniques employed in the past have been extremely varied: harvesting, separating, refining, retting, and decorating [108]. The physicochemical processing method, created by Bredemann in 1942 [61], can be used to extract fiber because it enables the measurement of the amount of pure fiber together with the HPLC method for the analysis of its phenolic compounds [61]. According to research on epoxy composites reinforced with nettle fibers, silane-treated fibers have a higher flexural strength than untreated ones [109]. Nettle fibers’ qualities are also improved by chemical treatments like those with sodium chlorite, hydrogen peroxide, and NaOH, which increase their suitability for composite applications. Increased tenacity, elongation at break, and crystallinity are the outcomes of these treatments [110]. Additionally, fibers treated with microwaves show higher tenacity when compared to those treated with conventional (alkaline) and ultrasonic methods [111]. Nettle/polyester composites have been found to perform better than neat polymers in terms of mechanical properties, including impact strength, tensile strength, and hardness [112]. Furthermore, compared to pure polyethylene, hybrid composites made of wool and nettle fibers mixed with low-density polyethylene (LDPE) exhibit greater flexural and tensile strength [113]. An appropriate aboveground biomass source for some natural products, stinging nettle shows the most promise for use in the pharmaceutical, feed, cosmetic, fiber and feed industries. Traditional folk uses of stinging nettle have been scientifically validated by the discovery of several active compounds in the plant [105].

### 3.4. Nettle Utilities 

**Figure 3:**
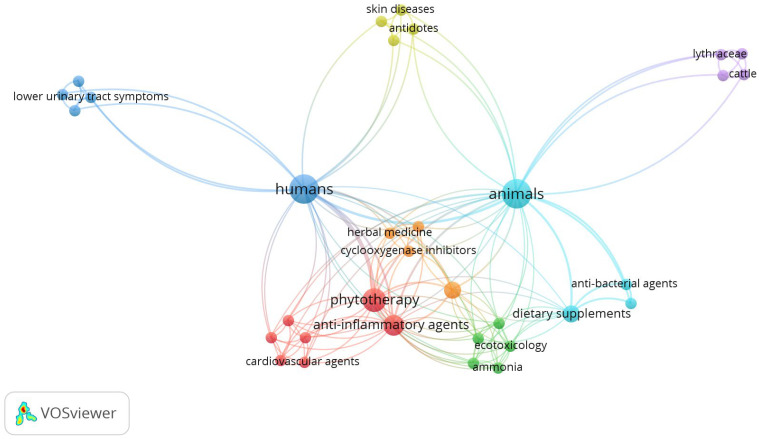
Map of key nettle utilities [25]: principal nodes as main concepts and links between them.

The plant known as stinging nettle has several therapeutic uses for both people and animals. Figure 3 draws attention to various usages by grouping them into many thematic groupings. These conceptual groupings are intimately connected to one another, demonstrating the variety and complexity of nettle’s use in many settings [114,115]. In the figure, the nodes represent concepts, with variable sizes correlated with their importance. Moreover, the links between nodes show the connections and their strengths. The main cluster includes general concepts, such as “humans” and “animals”, which are connected to the other topics. The colored links between clusters indicate how these relate to each other and what links exist between them according to the distance.

The first group worth noting is the blue cluster, which refers to the use of nettle by humans. Stinging nettle is a useful remedy for common lower urinary tract problems due to its diuretic and anti-inflammatory properties. Other important concepts in natural medicine are included in this category, such as phytotherapy, which describes the use of nettle in herbal remedies [116]. 

Phytotherapy, demonstrated by the red cluster, is one of the main sections of the study of nettles. It is an anti-inflammatory drug that works by blocking the enzyme cyclooxygenase, which is part of inflammatory processes. Nettle also promotes heart health and protects the circulatory system from ailments. This group emphasizes the use of nettle in humans and animals, comparing it to its use in humans [117].

In addition to phytotherapy, nettle is used in nutrition, ecotoxicology and other purposes. The green group represents studies describing the use of nettle in agriculture to reduce the effects of harmful substances such as ammonia. In addition, when used as supplementary food for humans and animals, it functions as a natural source of nutrients and improves overall health. The many uses of nettle in protecting the environment and animal health are highlighted by these links between toxicity and nutrition [118].

The yellow cluster is another of note, including studies describing stinging nettle as an antidote to various poisonous substances and its potential to treat skin conditions. Despite the fact that most of these applications are used in the veterinary industry, there may also be uses for humans. There is a link between nettles and the fields of ecotoxicology and food supplements, showing how important they are to animal health and care [119,120].

The image is completed by the purple cluster, which links nettle to its use in agriculture, especially in the care of cattle. It can be used to improve animal nutrition and as a treatment for a variety of diseases. 

Nettle is a complete solution with numerous agricultural and veterinary benefits, as demonstrated by the relationship between this group and those related to phytotherapy and toxicology [28,121]. 

In agriculture, nettle manure, also known as nettle slurry, is recognized as a useful organic fertilizer. This solution, which is derived from soaking nettles in water to extract their beneficial compounds, is widely used because of its nutrient-rich properties, especially because it stimulates plant growth. It is particularly effective due to the fact that nettles contain a large amount of minerals and trace elements, such as iron, potassium and nitrogen, which are vital for plant health. Because nettle litter is biodegradable and improves soil health without adding harmful chemicals, it is a sustainable farming practice. The European Food Safety Authority states that nettle helps in a greener approach to crop management and is considered an essential ingredient in agricultural practices [122].

Nettle slurry has been shown to be effective both as a growth promoter and as a natural pest repellent. Nettles, especially their alkaloids and flavonoids, are a natural remedy for fungal infections and insects. The use of nettle mud to improve plant vitality is supported by studies, which have shown it to be effective in organic farming systems. Agricultural practice with nettle products provides an example of how natural resources can be used to improve plant health and promote environmental sustainability [123].

## 4. Relation with Circular Bioeconomy

The European Union’s Bioeconomy Strategy focuses on sustainable and circular production, as well as on the conversion of different biological resources [124]. Many strategies for lowering the production of many items have been examined in order to transition from the linear economy to the bioeconomy and circular economy [125]. The primary challenge is transforming the present consumption patterns—which are mostly based on a production–use–waste paradigm—into a circular model, which includes developing new utilities or reusing existing ones. According to Kirchherr [126], investing in innovative environmental protection technology is essential for both humans and the circular economy. For most nations’ industrial sectors seeking long-term growth, the circular economy is a crucial subject [126,127,128,129,130]. A novel ecological model that uses CE approaches to encourage development is referred to by the term “CE” in certain research articles [131,132,133,134]. Many studies on the less acceptable behaviors as well as the limitations of the CE in regard to global sustainability have been carried out recently [135,136].

Stinging nettle (*Urtica dioica* L.) is frequently studied because it may be used as a feed supplement for poultry or as a high-yielding multifunctional feedstock [137,138,139]. Stinging nettle is a promising crop for nonfood markets, including the construction, paper, textile, and bioenergy sectors, and it grows well in Central Europe [138]. As was previously noted, nettle may be a good plant species to help reduce greenhouse gas emissions into the atmosphere. Compared to fully grown forests, it consumes substantially more atmospheric CO_2_ (18.8 t/ha) relative to biomass [61]. According to certain writers [139], this plant species is not cultivated enough in Europe for commercial purposes, being seen as a ruderal plant, but it is a promising candidate for numerous industry areas. General experimental plots encompass the current farmed regions. There are several uses for common nettle in the pharmaceutical, cosmetic, and agri-food industries [139].

There are now a number of commercial goods available on the market, such as soaps, shampoo to manage dandruff and enhance hair condition and lotions to cleanse the skin [41,106,138]. For many years, many extracts, whether aqueous or hydro-alcoholic, have been utilized to treat anemia [140,141]. Products from this plant species are used to treat a number of additional medical conditions, such as rheumatism, gout and issues with the kidneys, bladder or urinary system [11,142].

It is rather simple to transform nettle from a common and opportunistic plant to a helpful one. Nettle can be used in the automotive industry as a composite, as a textile fiber substitute for linen or hemp, or as a carbon or glass fiber substitute [143]. Fiber nettle is a developed kind of wild nettle. The vascular fibers of common nettle are silky, long, hypolignified and have a high tensile strength. The textile sector finds this trait to be quite appealing. The fibers at the base of the nettle stem have a cellulose concentration of about 80% and a lignin content of approximately 3.5–4.4%. Selection has increased the total fiber content, which in some cultivated varieties has reached up to 17–18% of stalk dry matter, compared to around 5% in wild nettle plants [137,138]. Additionally, it is well known that farmed plants with a higher density generate more fiber [137]. Unquestionably, in order to promote a sustainable, low-carbon bioeconomy, plant biomass must be used as an energy feedstock and source of (macro) molecules for industrial uses. Because they generate biomass more quickly than woody species, herbaceous crops are significant renewable resources. Plants that produce bast fibers, which are classified as fiber crops, are very desirable among herbaceous crops because they yield strong, long fibers that are rich in crystalline cellulose. In addition to their application in the textile industry, these fibers are valued by the biocomposite industry as environmentally benign substitutes for synthetic fibers [144]. 

A number of novel nettle clones have been chosen and identified based on their robust tillering and high fiber content [145]. In some EU nations, only fiber nettle clones have been used for experiments [138,139]. Under boreal growth circumstances, nettle’s yearly dry matter yield (DMY) varied between 6 and 10 Mg ha^−1^ [11], while the DMY of five clones’ stalks varied between 2.3 and 9.7 Mg ha^−1^ [138]. Fiber nettle clones have been successfully studied as a crop for bioenergy, primarily for anaerobic digestion [11]. Due to their lower CO_2_ emissions and lack of direct conflict with food production, some other crops that generate energy and can be grown on different soils also have a good effect on public acceptance of biofuel technologies [145,146,147]. However, due to the significant fiber content, there appears a strong resistance to bacteria during digestion that takes place without oxygen, and the majority of alternative feedstocks have slower anaerobic digestion rates and lower methane potentials than maize silage [146]. Because of this, using these feedstocks is still more costly than using corn silage.

A bioeconomy idea was created to address the issue of costly biogas plant feedstocks [148]. This concept involves thermochemically pretreating energy crops and leftover materials using steam explosion (SE) and then separating the solids from the liquids. While the aqueous part includes structures that decompose quickly and readily in a process of digestion that is considered anaerobic, the solid fraction is mostly composed of fibers, which are barely edible in the biogas process. The liquid is therefore used in biogas plants to generate heat and energy, while the solid part will be used as fiber material in various operations, including the production of packaging materials.

The most widely utilized and visible organic biomass worldwide is lignocellulose, which is also a crucial feedstock for biodegradable products, bioenergy technology and bio-based chemicals in biorefineries [147]. The microfibrils, which contain hemicellulose-linked cellulose included in a matrix of glycosylated proteins together with another polysaccharide, including lignin, make up its complex structure [149].

SE is one of these promising physicochemical techniques that enhances the accessible surface area of cellulose without significantly degrading it, breaks down the cell wall, eliminates hemicellulose and chemically alters the biomass all at once [150]. SE is a desirable technique for pretreating lignocellulosic materials since it can also effectively remove uronic and acetic groups autocatalytically, generating their correspondent acids and hemicellulose, which is depolymerizing [149,150,151,152,153,154]. SE is typically run between 160 and 220 °C and between 0.6 and 1.0 MPa of pressure. The lignin structure is broken down by boiling and quick depressurization, which also breaks down the hemicellulose into sugars and oligomers. Hemicellulose is depolymerized at pressures of around 0.5 MPa, whereas cellulose is not [155,156,157]. Moreover, it is simple to separate the liquid and solid phases after SE. Numerous studies have already investigated pretreatment of a fiber-rich substrate to increase the production of biogas [158,159,160,161].

**Figure 4:**
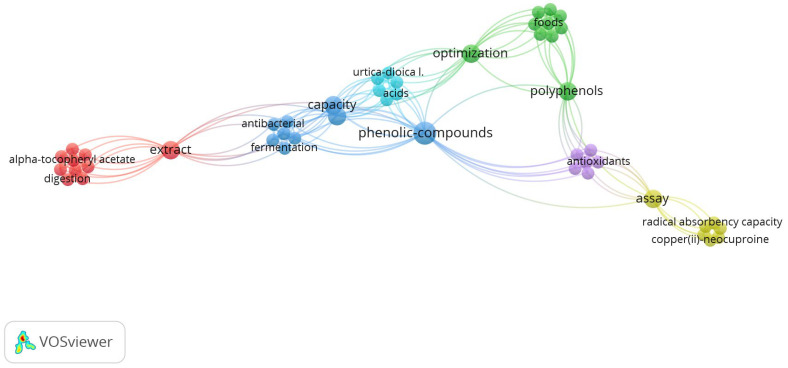
Map of key nettle relations with the circular economy [25]: principal nodes as main concepts and links between them.

Figure 4 illustrates the interconnectedness of nettle applications within the circular economy. Each node represents a research concept or term, such as ‘phenolic compounds’, ‘extract’, ‘antioxidants’, or ‘foods’, the size of which indicates their importance. “Phenolic compounds” is the central node, connected with all the other themes. The clusters represent key domains: red (bioactive compounds in digestion), blue (phenolic compounds in antibacterials), green (process optimization), and yellow (antioxidant evaluation). These connections highlight nettle’s potential to reduce waste and enhance resource efficiency.

The red cluster emphasizes antioxidants such alpha tocopherol acetate, a kind of vitamin E, and concentrates on extracts and digestive processes. These compounds, which are taken from plants like nettle, are utilized in digestive processes or as nutritional supplements. By removing these substances from vegetable waste or by-products, the circular economy may minimize waste and make better use of the resources that are available [162,163]. 

The blue group studies phenolic chemicals and how they can be used for fermentation and antibacterials. Using their antibacterial and antioxidant properties, phenolic compounds can be used to extend the life of products or can be used in industrial processes that require biological recycling. These molecules decrease the demand for synthetic chemicals by prolonging product life and preventing premature deterioration, emphasizing the circular economy [162,163].

The green cluster emphasizes the need for boosting efficiency in food and industrial production through process optimization and the use of polyphenols in food [164].

Antioxidants and their assessment via different assays are the main topics of the purple cluster. The quality of recycled or repurposed items depends on the capacity to quantify the antioxidants’ efficacy. In sectors like food and cosmetics that depend on the repurposing of natural substances, this kind of study facilitates quality management and monitoring [165,166].

Finally, the yellow cluster—which focuses on techniques to evaluate free radical scavenging capacity—is crucial to comprehending the antioxidant qualities of substances derived from nettles. This evaluation aids in determining the sustainability and quality of items in the circular economy, enabling them to be optimized for extended usage without endangering natural resources. 

## 5. The Novelty of This Study and the Positioning of Nettle in Relation to Other Bioeconomic Crops

Fiber crops represent an important category of economically valuable plants due to their diverse industrial applications, including their use in composites for construction and automotive industries, as well as in textiles, paper, mats, hats, ropes and cordage. Other crops like kenaf, hemp and flax have also been researched for these purposes. Utilizing fiber crops, in general, including those from nettle, for energy and biomaterials could contribute to reducing fossil fuel dependence. Each application requires a comparative assessment with synthetic or mineral alternatives to evaluate the energy efficiency of fiber production and consumption. However, there remains a shortage of comprehensive life cycle assessments in the fiber crop literature, with most studies focusing on flax or hemp [167,168]. 

In comparison to secondary fibers, primary fibers are bigger and longer. The cell walls of secondary fibers are highly lignified and are thinner and shorter. Because of these qualities, nettle fiber usage in textiles could be an advantage [169]. Secondary fibers are mostly utilized for cordage, pulp and recycling additives because of their properties [170]. As the plant ages, the amount of secondary fibers grows and decreases along the stem. Plant harvesting timing is crucial to increasing the amount of fiber extracted, since the characteristics of bast fiber vary as plants age because of the growing amount of secondary fiber [171]. In addition to its industrial applications, nettle offers potential health benefits that distinguish it from other fiber crops. For instance, nettle can serve as a source of bioactive compounds, similar to flaxseed, which is used in foods, medicines, and as a feed ingredient. Nettle also contains a variety of beneficial nutrients and phytochemicals, which could support additional bioeconomic uses, ranging from nutraceuticals to natural additives in cosmetics. Comparatively, flaxseed and linseed oil are already utilized in various industries, including food, pharmaceuticals, and oleochemicals, where they are used to produce paints, soaps, and biodiesel [172]. There is potential for producing fiber from stinging nettle grown on soils polluted with trace elements. In comparison to regions that were not contaminated, the nettles that were growing in poplar short rotation coppices had lower amounts of pollutants in their fibers. Nettles are a potential addition to European fiber crops for material applications, even if their biomass production is lower than that of conventional crops. The fibers’ tensile qualities were on par with or superior to those of industrial hemp and flax [173].

## 6. Conclusions

This study reveals that nettle processing has significant potential within circular economy frameworks, offering solutions for sustainable resource use and reduced ecological impact. The plant’s adaptability and low ecological requirements make it ideal for production in diverse environments, including marginal lands that areunsuitable for food crops. Nettle’s bioactive compounds support multiple sectors, from health to material sciences, and its fibers provide a renewable alternative to traditional materials. In conclusion, nettle represents a promising asset for advancing sustainable practices, necessitating further research and investment in its large-scale cultivation and application across industries. Future work could focus on optimizing extraction methods and exploring additional commercial uses within the bioeconomy.

Because the research is based on a review of the literature, the quality and availability of data from earlier research will affect this study’s findings. The correctness of the results may have been impacted by several significant sources that were not completely available or that lacked complete methodological information. The limitations on the economic assessment are as follows: There are not many analyses of the financial advantages and disadvantages of nettle farming. This study places more emphasis on the theoretical possibilities than on the specific financial elements, which might affect its usefulness in different sectors.

Although nettle has the potential to be used in pharmaceutical and cosmetic goods, this study offers insufficient information on the long-term safety and biocompatibility of nettle extracts. Key limitations include the lack of comprehensive economic assessments for nettle farming and the absence of long-term safety data for pharmaceutical applications. Collaborative frameworks involving academic and industrial stakeholders could address these gaps. Emerging technologies, such as CRISPR-based crop improvement, present opportunities to enhance nettle’s yield and resilience, although these require significant research investment and regulatory approvals.

## Figures and Tables

**Table 1:** Nettle main bioactive compounds, extraction methods and effects.

Chemical Group	Compounds	Extraction Method	Properties	References
Phenolic acids	Gallic acid	Ultrasound-assisted extraction method (UAE)	Antioxidant, anticarcinogenic, anti-inflammatory, apoptosis inducer.	[56,57]
	Vanillic acid	Gas Chromatography–Mass Spectrometry (GC-MS) method	Anti-obesity, anti-inflammatory, anticarcinogenic, antibacterial.	[48,58]
	Caffeic acid	Maceration method	Antioxidant, cosmetics (collagen inducing), antimicrobial.	[59,60]
	Chlorogenic acid	High-performance liquid chromatography (HPLC) method	Antiplatelet action.	[61]
	Caffeoyl malic acid	High-performance liquid chromatography (HPLC) method	Antiplatelet action.	[61]
Flavonoids	Catechin	Aluminum chloride method	Antioxidant, anticarcinogenic, neuroprotective, anti-cardiovascular diseases, hepatoprotective.	[62,63]
	Kaempferol	Soxhlet method	Anticarcinogenic, anti-inflammatory.	[64,65]
	Isorhamnetin	Ultrasound-assisted extraction method (UAE)	Antiviral, antioxidant, anti-tuberculosis, anti-inflammatory.	[64,66]
Amino acids	Alanine	Gas chromatography–mass spectrometry (GC-MS)	Blood sugar regulation	[67,68]
	Glutamic acid	Thin-layer electrophoresis and chromatography method	Anti-carcinogenic	[69,70]
	Leucine	Liquid chromatographic method	Insulin secretagogues, anti-sarcopenia, anti-type 2 diabetes.	[71,72]
	Isoleucine	High-performance liquid chromatography (HPLC) method	Protein metabolism, fatty acid metabolism, glucose transportation, immunity.	[73,74]
	Histidine	Spectral methods	Protein synthesis, antioxidant, neurotransmitter.	[75,76]
	Lysine	Aqueous two-phase system (ATPS) method	Anticarcinogenic, antitumor.	[77,78]
	Methionine	Gas chromatography (GC) method	Metabolic process regulator, anticarcinogenic, anti-neurodegenerative diseases.	[79,80]
	Phenilalanine	Aqueous two-phase system (ATPS) method	Antidepressant agent.	[81,82]
	Threonine	High-performance liquid chromatography with UV (HPLCUV) method.	Antispastic.	[83,84,85]
	Valine	Aqueous two-phase systems (ATPSs) method	Antimicrobial, immunity booster.	[86,87]
Carotenoids	β-Carotene	Reverse phase High-Performance Liquid Chromatography (HPLC)	Antioxidant.	[40,85]
	Neoxanthin	Reverse-phase High-Performance Liquid Chromatography (HPLC)	Anticarcinogenic, chemo-preventive, antioxidant.	[40,88]
Organic acids	Acetic acid	Ultrasound-assisted extraction (UAE)	Anti-cardiovascular diseases, anticarcinogenic.	[89,90]
	Citric acid	Ultrasound-assisted extraction (UAE)	Acidity regulator, antioxidant, antibacterial.	[89,91]
Fatty acids	Arachidic acid	Rapid extraction system for solid–liquid extraction (Soxtherm) method	Potential protective role against Schistosoma Mansoni https://www.sciencedirect.com/topics/medicine-and-dentistry/schistosoma-mansoni accessed on 15 December 2024, *S. haematobium* infection and tumor initiation.	[92,93]
	Behenic acid	Rapid extraction system for solid–liquid extraction (Soxtherm) method	Obesity averter	[92,94]
Chlorophyll	Chlorophyll E140	High-Performance Liquid Chromatography (HPLC)	Green coloring agent	[95,96]

## Data Availability

Data are contained within the article.
